# Mindfulness Practices for Children and Adolescents Receiving Cancer Therapies

**DOI:** 10.1177/27527530211056514

**Published:** 2022-01-21

**Authors:** Shelley Murphy, Ani Jamyang Donma, Sara Ahola Kohut, Elli Weisbaum, Jacqueline H. Chan, Erin Plenert, Deborah Tomlinson

**Affiliations:** 1 Department of Curriculum, Teaching and Learning, 7938 University of Toronto, Toronto, Ontario, Canada; 2 Spiritual & Religious Care Department, 7979 The Hospital for Sick Children, Toronto, Ontario, Canada; 3 Child Health Evaluative Sciences, 7979 The Hospital for Sick Children, Toronto, Ontario, Canada; 4 Department of Psychology, 7979 The Hospital for Sick Children, Toronto, Ontario, Canada; 5 Department of Psychiatry, 7938 University of Toronto, Toronto, Ontario, Canada; 6 Department of Neuroscience and Mental Health, 7979 The Hospital for Sick Children, Toronto, Ontario, Canada; 7 Institute of Medical Science, Faculty of Medicine, 7938 University of Toronto, Toronto, Ontario, Canada

**Keywords:** mindfulness-based intervention, body scan, trauma-sensitive, alternative therapies

## Abstract

**Background:** Mindfulness is our innate capacity to pay full, conscious, and compassionate attention to something in the moment. It is also a skill that can be strengthened by mental practice. More recently, mindfulness-based interventions (MBIs) are identified within clinical practice guidelines as an intervention in the treatment of certain symptoms for children with cancer. However, there is little guidance available on the practice of using MBIs in the pediatric oncology population. The aim of this paper is to provide an overview of mindfulness, highlights symptoms where mindfulness practices may be of benefit, identifies trauma-sensitive considerations, and provides examples of MBIs that may be considered in the context of pediatric oncology. **Methods:** Collaboration of expert opinion, which included The Mindfulness Project Team, has enabled this collective informative paper. **Results:** Mindfulness has been recommended to help with the symptom of fatigue in children with cancer. Emotional symptoms such as anxiety, sadness, and anger may also benefit from the use of MBIs. Ideal MBIs for this population may include mindful movement, mindfulness of the senses, mindfulness of breath, mindfulness of emotions, and the body scan. These approaches can easily be adapted according to the age of the child. Many approaches have been administered with minimal training, with very few requiring a facilitator. However, hospitals have started to incorporate mindfulness experts within their care provision. **Conclusion:** Future research should continue to investigate the use of MBI programs for children with cancer.

## Background

Increases in childhood cancer survival rates are due, in part, to more intense treatment protocols. As a result, children receiving chemotherapy are severely bothered by many symptoms and experience these throughout their cancer journey (Hyslop et al., 2018; Johnston et al., 2018; Tomlinson et al., 2019). Management of these symptoms in children is evolving and, for certain symptoms, includes complementary and alternative medicine (Zucchetti et al., 2019).

Mind and body practices have been introduced within healthcare practice to assist with issues such as depression, anxiety, and chronic pain (Creswell, 2017). Mindfulness has also been introduced in schools to help students with their focus, cognition, executive functioning, and social–emotional learning (Bostic et al., 2015; Feuerborn & Gueldner, 2019; Geronimi et al., 2020; Schonert-Reichl et al., 2015). In a recent systematic review of mind and body, practices used to treat fatigue in patients with cancer or hematopoietic stem cell transplant (HSCT) mindfulness and relaxation were found to be effective (Duong et al., 2017). Consequently, a clinical practice guideline for the management of fatigue in children and adolescents with cancer and in pediatric recipients of HSCTs strongly recommended the use of physical activity, relaxation, and mindfulness to reduce fatigue, supported by evidence of moderate quality (Robinson et al., 2018).

We have previously identified mindfulness-based interventions (MBIs) that have been used with children to manage symptoms associated with cancer therapy to describe what is known about this modality of treatment in this population of children (Tomlinson et al., 2020). The six studies included in this review examined the impact of MBIs on procedural pain and on a variety of psychological symptoms including distress and coping. Interventions mainly consisted of breathing exercises and meditation practices. Despite mindfulness practice requiring attentional control, focus, and cognitive concentration, even children as young as 4 years of age have been able to be engaged (Burke, 2010).

Although recognition that MBIs could be used to help in the treatment of certain symptoms in children and adolescents receiving cancer treatment, there is a lack of clarity about the types and schedules of MBIs that may be used effectively for this population. This article aims to: (1) provide an overview of mindfulness, (2) highlight symptoms where mindfulness practices may be of benefit, (3) identify trauma-sensitive considerations, and (4) provide examples of mindfulness-based approaches that may be considered in the context of pediatric oncology.

## Mindfulness

Mindfulness is our innate capacity to pay full and conscious attention to something in the moment. As a mental state or trait, it is the quality of presence we bring to everything we do. It is often defined as, “the awareness that emerges through paying attention on purpose, in the present moment, and nonjudgmentally to the unfolding of experience” (Kabat-Zinn, 2003, p. 144).

Mindfulness practice, which has its roots in Buddhism and other contemplative traditions that trace back thousands of years, is a means through which states and eventual traits of mindfulness are pursued and cultivated. As such, mindfulness is a skill that can be strengthened through practice. More recently, mindfulness practice has come to the forefront in the West as an approach within medical science. Most notably, Kabat-Zinn developed an 8-week program called Mindfulness-based Stress Reduction (MBSR) at the University of Massachusetts Medical Center in the late 1970s. MBSR was developed as a secular health intervention and has been shown to be successful in treating medical conditions including depression, anxiety, chronic pain, heart disease, and cancer (Baer, 2015).

### Mindfulness and Fatigue

Fatigue is generally defined as a feeling of lack of energy and motivation that can be physical, mental, or both. Fatigue is not the same as drowsiness, but the desire to sleep may accompany fatigue. Apathy is a feeling of indifference that may accompany fatigue or exist independently. In addition, children with cancer often describe fatigue using a variety of terms including tiredness, weary, loss of strength, dizziness, feeling drained, feeling drowsy, lacking motivation, exhaustion, and feeling emotional (Tomlinson et al., 2016).

Cancer-related fatigue affects the quality of life of pediatric (Grace et al., 2020) and adult patients (Stadtbaeumer et al., 2020) which can contribute to depression and pain. It has been shown that mindfulness practices can have an impact on the quality of life of pediatric (Tomlinson et al., 2020) and adult cancer patients (Dehghan et al., 2020).

Fostering peace of mind that can include openness, compassion, acceptance, training concentration, and nonjudgment are foundational components of mindfulness practice. By treating the whole person, it targets not just fatigue, but many of the secondary cognitive and socio-emotional factors associated with fatigue such as stress, reduced ability to focus, sense of groundlessness, or entrapment. Mindfulness exercises that incorporate slow and gentle breath training or movement forms such as Qi gong or Tai chi also have therapeutic effects (Wayne & Fuerst, 2013). Mindfulness trains heightened body and self-awareness, with compassion rather than judgment or overthinking. Training heightened self-awareness of bodily sensations helps an individual sense the inner landscape of the body and better distinguish areas that may be weak or strong, tense or relaxed, or more or less tired. This awareness can play a key role in the prevention and rehabilitation of different medical conditions such as cancer (Wayne & Fuerst, 2013). Mindfulness training can be a safe and kind therapeutic guide that can act as an inner compass for rehabilitation and recovery. Developing inner sensitivity can potentially help the patient learn to identify the feelings and sensations in the body and communicate these feelings to caregivers. Greater inner awareness to the body also helps facilitate attention to posture, excessive strain in the muscles, bringing attention to restricted breathing, and fostering more inner sensitivity (Wayne & Fuerst, 2013).

### Mindfulness and Emotions

Children with cancer often have significant social–emotional challenges resulting not only from the illness itself but from anxieties associated with diagnosis and prognosis, side effects of treatments, and hospital stays resulting in separations from familiar settings and social supports (Abedini et al., 2021). Furthermore, when children are confronted with unfamiliar or difficult emotions such as anger, fear, guilt, or denial it can become increasingly difficult for them to manage their stress response.

Several studies have shown mindfulness to increase an individual’s capacity to observe and experience strong emotions with greater objectivity and less reactivity (Lippelt et al., 2014; Shapiro et al., 2006; Villamil et al., 2019). Purposefully and repeatedly facing one’s discomforts and fears is a technique used to treat a variety of disorders and is often referred to in the literature as *exposure* (Barlow et al., 2000). In their seminal work on the mechanisms of mindfulness theory, Shapiro and colleagues (2006) propose that *exposure* is a key outcome activated through the development of mindfulness. The authors suggest it is through direct exposure that one learns to experience emotions, thoughts, or body sensations as less overwhelming or frightening by cultivating a different relationship to them (Shapiro et al., 2006). In mindfulness, this exposure occurs in the context of kind and compassionate awareness. This compassionate stance is a key ingredient to support successful exposure.

Mindfulness practices have the potential to give children an opportunity to become skillful at being aware of their emotions as they arise in the present moment and familiar with identifying, understanding, using, and managing them. Children learn to become aware of the connection between thoughts, emotions, and bodily sensations (Murphy, 2019). A landmark study conducted by UCLA professor of psychology, Lieberman and colleagues (2007) found that *affect labeling* (putting feelings into words) supports emotion regulation, particularly for more difficult emotions such as sadness and anger. Putting feelings into words helps reduce their intensity by activating the prefrontal region of the brain (thinking and reasoning part of the brain) and quieting the amygdala (the stress response). This is of great consequence for children who are dealing with symptoms, stressful states, and negative emotions connected to their experience with cancer.

## Trauma-Sensitive Considerations

Research has shown that within the first few years after diagnosis, cancer-related post-traumatic stress symptoms (PTS) are quite common in children with cancer. Typically, symptoms resolve and distress levels return to baseline. However, a significant subset of children with cancer will continue to experience persistent traumatic stress reactions in the form of cancer-related PTS and PTS disorder for years after treatment has ended (Marusak et al., 2019). As a result, there has been a growing emphasis on optimizing psychosocial outcomes, particularly through more extensive early intervention. Mindfulness has shown early promise as one such intervention.

Mindfulness has been shown to help mitigate the negative consequences of traumatic stress experiences by strengthening resilience (i.e., successful management and coping with ongoing experiences of stress and adversity) and by influencing the related physiological effects of stress (Ortiz & Sibinga, 2017). Furthermore, mindfulness programs can improve coping, resiliency, and quality of life in patients with depression, anxiety, and cancer (Fish et al., 2014). Collectively, studies have suggested that mindfulness has the potential to buffer the effects of stress and trauma in children and into adulthood (Ortiz & Sibinga, 2017).

It is important to note that while mindfulness has been shown to help protect against and counteract traumatic, recurrent, and/or prolonged stress experienced through cancer and treatment, it should be introduced through a trauma-sensitive lens. This is because when mindfulness is introduced without an awareness of trauma, traumatic stress symptoms can be triggered or exacerbated (Treleaven, 2018). As mindfulness and trauma expert, Treleaven has suggested, mindfulness will not cause trauma, but it can reveal it (Treleaven, 2018). This becomes particularly salient as someone may be triggered out of what is known as their window of tolerance as a result. Coined by Siegel (1999), a clinical professor of psychiatry, the window of tolerance describes stability of the nervous system (a capacity to manage challenges and stresses in the moment) as opposed to dysregulation (i.e., hyperarousal or hypoarousal). Trauma-sensitive considerations help ensure that children are not exceeding what they can handle in practice. It is therefore important to ensure that mindfulness instruction is delivered by trained individuals and with collaboration between trauma-informed clinicians and educational teams (Schmidt & Lawson, 2018).

Examples of trauma-sensitive mindfulness considerations for providers are: (1) Offer choice and modifications for practices; (2) Be clear about what participants should expect in terms of length of practice, type of practice, etc.; (3) Know the signs of trauma in order to recognize and be responsive them; (4) Incorporate movement to make it easier for trauma survivors to stay present with sensations; (5) Offer choice in whether to take breaks or practice at all, have eyes opened or closed, whether to sit, stand, or lie down during practice, and which anchor to use for attention; and (6) Recognize that the breath is not always neutral (Treleaven, 2018). When mindfulness is offered with an awareness of trauma, it helps reduce the risk of exacerbating symptoms of traumatic stress. It also makes it much more likely that mindfulness participants will benefit from its power (Treleaven, 2018).

## Mindfulness-Based Practices

The two most utilized mindfulness-based clinical interventions in oncology are MBSR and mindfulness-based cognitive therapy (Cramer et al., 2016). However, more brief and less intense formats of MBI have been utilized as well. Mindfulness-based activities, such as mindful movement, mindfulness of the senses, mindfulness of breath, mindfulness of emotions, and the body scan, can be adapted well for different age groups. These activities are discussed below and summarized in Table 1.

**Table 1.** Mindfulness-Based Practices Feasible for Use in Children With Cancer.

Mindfulness-based practice	Description of practice	Examples
Mindful movement	Invites children to bring awareness to the connection between their bodies and minds through focused attention and a repeated linking of their micro-movements with their breath	Mindful walking that focuses attention on the breath and the slow, intentional micro-movements of the upper body, legs, and feet with each step Mindful yoga (chair yoga, tree pose, warrior pose, etc.) Mindful mirroring which is a partner activity where one partner leads gentle, slow and deliberate body movements while the other partner mirrors these
Mindfulness of the senses	Purposeful attention to the present moment through one or a combination of the five senses	Five-senses tour that uses purposeful attention to whatever comes into awareness through each of the available senses Mindful listening which focuses awareness on the coming and going of sound in the environment Mindful seeing which is observing surroundings and noticing, with thoughtful attention, what captures visual attention Mindful eating that directs mindful attention to something being eaten using each of the senses
Mindfulness of breath	Purposeful attention to the natural flow of the in-breath and out-breath as an anchor or home base for the present moment	Mindful breathing by consciously focusing on the natural rhythm and pace of the breath and returning to it when the mind wanders 5-finger breathing by focusing attention on the breath for 5 cycles of in and out breathing, tracing each finger for each breath Square breathing where the pattern of breathing is symbolized by a square or box.
Mindfulness of emotions	Noticing feelings and putting them into words	Emotion mapping where children are invited to write about or illustrate emotions and where they are feeling these emotions within their bodies Worry box activity where children are invited to put their worries in images or in writing and then place them in a container specifically designed to hold them with kindness Gratitude practice where children are invited to think about and name or record 2 or 3 things that they are grateful for each day and why they are grateful for these things
The body scan	A guided practice in which children are invited to bring heightened awareness to the body in a sequential and structured way	Children are guided to use their awareness to take a tour of their bodies with curiosity and kindness/friendliness

### Mindful Movement

Mindful movement is an active mindfulness practice that invites children to bring awareness to the connection between their bodies and minds through focused attention and a repeated linking of their micro-movements with their breath (Murphy, 2019). Research has connected this type of practice with improvements in stress management, self-awareness, cognition, and positive affect (Thygeson et al., 2010; Zelazo & Lyons, 2012). Examples of mindfulness-based movement activities are mindful walking (consciously focusing attention on the breath and the slow, intentional micro-movements of the upper body, legs, and feet with each step as one walks); mindful yoga (chair yoga, tree pose, warrior pose, etc.); mindful mirroring (a partner activity in which one partner leads gentle, slow and deliberate body movements while the other partner mirrors these movements.) (Murphy, 2019) and Qigong. Qigong (or Chi gong) is a system of coordinated gentle exercise and relaxation through meditation and breathing exercise based on the Chinese medicine theory of energy channels (Oh et al., 2008).

### Mindfulness of the Senses

Mindful sensing is bringing purposeful attention to the present moment through one or a combination of the five senses: sound, sight, touch, taste, and smell. This type of practice strengthens the ability to direct attention and gently redirect attention when confronted with distraction. A part of the brain called the reticular activating system (RAS) controls the fight-or-flight system and acts as a filter for most of the incoming sensory input or data from our environment (Garcia-Rill et al., 2013). In part, the role of the RAS is to decide which data should be brought into the conscious mind and what information can be safely ignored. Mindful sensing strengthens the RAS through opportunities of intentionally directing mindful attention through our senses (Murphy, 2019).

Examples of mindful sensing practices are five senses tour (purposefully paying attention to whatever comes into awareness through each of the available senses); mindful listening (focusing awareness on the coming and going of sound in the environment); mindful seeing (observing surroundings and noticing, with thoughtful awareness, what captures visual attention); mindful eating (directing mindful attention to something being eaten [i.e., raisin, small piece of chocolate, etc.] by engaging all of the senses) (Murphy, 2019).

Mindful sensing practices can also be incorporated into everyday activities. Once initially introduced to mindfully eating a small piece of chocolate, children can then generalize those skills to mindfully eating a meal, or once introduced to mindful listening, children may begin to practice mindfully listening to others in conversation. Incorporating mindfulness into everyday activities can support ongoing practice, especially for children who may struggle to find time and space for formal practice.

### Mindfulness of Breath

Mindfulness of breath is an exercise in purposely paying attention to the natural flow of the in-breath and out-breath as an anchor or home base for the present moment. Focusing on the inhalation and exhalation of the breath allows thoughts, emotions, and sensations to come and go in the background (Murphy, 2019). The practice of mindful breathing has been shown to slow the body’s breathing pattern down, intensify vagus nerve action, reduce negative emotions, and regulate the body’s responses to stress (Chui et al., 2021).

Examples of mindful breathing exercises are mindful breathing (consciously focusing on the natural rhythm and pace of the breath and returning to it when the mind wanders); five finger breathing (a technique that gives visual and tactile cues to help focus attention on the breath for five cycles of in- and out-breathing. With the hand in a high-five position, the index finger of the opposite hand begins at the bottom of the thumb and traces up the outside while taking a breath in. It then traces down the inside of the thumb while simultaneously breathing out. This continues throughout the remaining four fingers for a total of five breath cycles.); Square breathing (this breathing exercise is named for the pattern of breathing symbolized by a square or box). There is an in-breath and an out-breath and two pauses in this practice. Each of these is meant to be the same length, just as each side of the square is the same length. It unfolds as breathing in for the count of 4, holding the breath for a count of 4, breathing out for a count of 4, and holding the breath for a count of 4 (Murphy, 2019).

### Mindfulness of Emotions

Mindfulness of emotions activities helps children to recognize, name, and manage their emotions. They also help children understand that emotions have a direct effect on the sensations they perceive within their body. Children who have high levels of stress have a much more difficult time recognizing and managing their emotions. Although children are often able to identify emotions when they are feeling them at their extreme, they are typically less able to notice and name them on the way to the extreme (Murphy, 2019). By noticing feelings and putting them into words, the prefrontal region of the brain is activated, and the stress response is quieted. This in turn helps to put the brakes on a negative emotional response (Lieberman et al., 2007). Through mindful emotional practices and activities, children become better attuned to their emotions and responses which leads to a greater likelihood that they will be able to manage and regulate them (Murphy, 2019).

Examples of mindfulness of emotions activities are emotion mapping (children are invited to write about or illustrate what emotions they are feeling and where they are feeling these emotions within their bodies); worry box activity (children are invited to put their worries in images or in writing and then place them in a container specifically designed to hold them with kindness). The intention of this activity is to help children bring awareness to what they are feeling—without ignoring or pushing their feelings away. If their emotions are particularly dysregulating, they may be invited to place them in a worry box. Its purpose is to provide a temporary, observable and kind distance between themselves and their worries should these worries begin to take them outside of their window of tolerance (Siegel, 1999); gratitude practice (children are invited to think about and name or record two or three things they are grateful for each day and why they are grateful for these things). Mindfulness of one’s emotional experiences is essential to well-being. When children become skillful at recognizing and naming what they are feeling at any given moment, they are more likely to be able to calm themselves in the face of challenging emotions and stress. Furthermore, those who practice mindfulness on a regular basis tend to be both physiologically and psychologically healthier, have fewer negative emotions, and have greater resilience (Emmons & Mishra, 2011).

### The Body Scan

The body scan practice, typically utilized in programs such as MBSR, is a guided practice in which children are invited to bring heightened awareness to the body in a sequential and structured way. It is considered a foundational mindfulness practice (Kabat-Zinn & Hanh, 2009). It provides an opportunity for children to strengthen their present awareness and become more attuned to the sensations in their bodies in a compassionate versus judgmental or critical way. Although many participants tap into greater states of calm and relaxation as a byproduct of participating in the body scan practice, these are not the primary goals. The goal is to train the mind to be more openly aware of sensory experiences as one shifts attention from one body part to the next and to do so without judgment (Kabat-Zinn & Hanh, 2009). This ultimately helps to cultivate more acceptance of what arises.

Within a body scan practice, children are guided to use their awareness to take a tour of their bodies with curiosity and friendliness. They are invited to notice each part of their body as it is in the present moment, without trying to change anything (whether pleasant, unpleasant, or neutral). Depending on age level, the length of the body scan can be modified (Murphy, 2019). For children with cancer, the body scan can be particularly supportive given their bodies can often be a source of pain and negative emotions. According to research, the body scan helps participants notice the pain they are experiencing without trying to change it. This detached awareness helps to shift the relationship to pain and to the body itself, which can ultimately support pain relief and reductions in stress (Carmody & Baer, 2008).

Prior to body scan, it can be helpful for children with cancer to first find a safe or neutral space to turn their attention to if the experience of the body scan becomes too intense. This safe space may be another area of the body (i.e., the feet which are typically neutral), or the breath itself as an anchor. This could enable children to increase the amount of time they are able to pay direct, nonjudgmental attention to sensations they may have previously been avoiding. Furthermore, this may help ensure they are able to stay within their window of tolerance in a trauma-sensitive way (Treleaven, 2018).

## Mindfulness Program for Adolescents

In one research study, participants took part in the Mindful Awareness and Resilience Skills for Adolescents (MARS-A) program (Vo, 2015) delivered both on-site and virtually through the Hospital for Sick Children, Toronto, Canada. These adolescents consistently reported that mindfulness practices helped them to observe and “*be with*” their pain and illness in a way that felt less overwhelming. For example, participants shared that Open Awareness practices, which focus on noting and monitoring thoughts and feelings as they arise, helped them to become objectively aware of feeling anxious about pain they were experiencing or a “negative” diagnosis they had recently received without feeling as overwhelmed by emotions such as fear, shame, or hopelessness that may accompany a diagnosis or experience in hospital. Adolescents in the MARS-A program often highlight the body scan as a favorite practice, noting that it helps them learn to offer kindness and compassion to pain in their body, while also learning to recognize and have gratitude for the parts of their bodies that are not experiencing pain. Although this outcome of a body scan practice is encouraging, facilitators should keep in mind that a body scan practice should be handled with care and guided with a trauma-informed lens. When we deliver the MARS-A program at the Hospital for Sick Children, we iterate the framing and guidance of a body scan to account for the needs of the specific population we are working with. For example, within the context of guiding a body scan with a population experiencing chronic pain or fatigue, prior to beginning the guidance we ask participants to identify a part of their body that can be their “safe space.” We then invite them if they feel uncomfortable or overwhelmed at any point during the practice to self-regulate by shifting their attention to their “safe space” as a way to calm their nervous system. After this, they can determine for themselves if they want to reengage with the guided practice or remain in their “safe space.” One adolescent with cancer reported that through this experience they were able for “the first time in a long time” to feel safe in their body and be “kinder” to their pain. These examples help to illustrate the potential for mindfulness to enhance patients’ capacity for *exposure*. Through this exposure, they can begin to shift their relationship to, and experience of, emotions related to their illness and pain. This can reduce the emotional fatigue related to their illness, which has the potential to enhance their overall sense of well-being. This in turn can increase their clarity and agency regarding their needs in relation to navigating their ongoing treatment and healing.

## Conclusions

Children with cancer often experience significant challenges not only from the illness itself but from the anxieties, fatigue, and emotional trauma associated with diagnoses, prognoses, and treatment. MBIs are an emerging therapy that can be incorporated to improve both physical and psychosocial well-being. Studying the effect of mindfulness may be hindered by several factors including the variety of practices that constitute mindfulness and the availability of trained facilitators. The activities listed would be simple and suitable for children with cancer.

Next steps in the introduction of MBI programs to children with cancer need to focus on feasibility studies (Tomlinson et al., 2020). Consideration needs to be given to who delivers instruction, collaboration between clinicians and educational teams (Schmidt & Lawson, 2018), and the success and sustainability of the program. With training, nurses have the potential to include these simple mindfulness activities into their practice. Nurses could incorporate mindfulness as a short daily activity or during occasions such as painful procedures, anxious, or painful episodes.

Future research could pilot the feasibility and use of mindfulness programs based on these suggested activities. With mindfulness being recommended as treatment within certain clinical practice guidelines and care pathways, it is vital that we develop practices that are trauma-sensitive and readily available to children with cancer and their families.
